# Supplementary material for: Sequencing of SARS-CoV-2 RNA Fragments in Wastewater Detects the Spread of New Variants during Major Events
Source: Microorganisms. 2023 Oct 30;11(11):2660. doi: 10.3390/microorganisms11112660 (PMC10672834; doi:10.3390/microorganisms11112660)
Supplement: Supplementary file 1 [file microorganisms-11-02660-s001.zip › microorganisms-2625135-supplementary.pdf]

Supplementary Materials

Table S1. Number of WEF 2023 participants per region and country of origin.

Africa	North Africa	Algeria	1
		Egypt	5
		Morocco	10
		Sudan	2
		Tunisia	2
	Eastern Africa	Ethiopia	2
		Kenya	11
		Mauritius	1
		Mozambique	2
		Rwanda	5
		Uganda	1
		Tanzania	5
		Zambia	5
		Zimbabwe	6
	Middle Africa	Angola	3
		Chad	1
		Democratic Republic of the Congo	2
	Southern Africa	Botswana	2
		South Africa	50
	Western Africa	Benin	1
		Ivory Coast	1
		Ghana	3
		Niger	2
		Nigeria	14
		Senegal	1
		Togo	1
Europe	Eastern Europe	Belarus	1
		Bulgaria	3
		Czech Republic	5
		Hungary	1
		Poland	6
		Moldova	1
		Romania	1
		Russian Federation	1
		Slovakia	3
		Ukraine	10
	Northern Europe	Denmark	25
		Estonia	1
		Finland	7
		Ireland	8
		Latvia	2
		Lithuania	4
		Norway	29
		Sweden	26
		United Kingdom	236
	Southern Europe	Albania	2
		Croatia	2
		Greece	5
		Italy	24
		Malta	1
		Montenegro	1
		Republic of North Macedonia	1
		Portugal	8
		Serbia	2
		Slovenia	2
		Spain	23
	Western Europe	Austria	10
		Belgium	25
		France	70
		Germany	119
		Liechtenstein	4
		Luxembourg	5
		Monaco	1
		Netherlands	58
		Switzerland	259
Americas	Caribbean	Dominican Republic	1
		Grenada	1
		Jamaica	1
		Trinidad and Tobago	1
	Central America	Costa Rica	5
		El Salvador	2
		Guatemala	3
		Honduras	2
		Mexico	15
		Panama	1
	South America	Argentina	7
		Brazil	47
		Chile	6
		Colombia	13
		Ecuador	6
		Paraguay	1
		Peru	5
		Uruguay	1
		Venezuela	2
	Northern America	Bermuda	1
		Canada	39
		USA	721
Asia	Central Asia	Kazakhstan	2
	Eastern Asia	People's Republic of China	38
		Hong Kong SAR, China	28
		Taiwan, China	2
		Republic of Korea	36
		Japan	80
	South-eastern Asia	Cambodia	1
		Indonesia	16
		Malaysia	10
		Myanmar	2
		Philippines	18
		Singapore	38
		Thailand	16
		Timor-Leste	2
		Viet Nam	3
	Southern Asia	Bangladesh	4
		Bhutan	1
		India	95
		Iran	1
		Nepal	3
		Pakistan	12
		Sri Lanka	1
	Western Asia	Azerbaijan	7
		Bahrain	11
		Cyprus	2
		Georgia	4
		Iraq	4
		Israel	11
		Jordan	7
		Kuwait	14
		Lebanon	2
		Oman	2
		Qatar	10
		Saudi Arabia	46
		Palestinian Territories	4
		Türkiye	14
		United Arab Emirates	75
Oceania	Australia/ New Zealand	Australia	16
		New Zealand	2
	Melanesia	Fiji	1

Table S2: Point estimates with upper and lower 95% confidence intervals (CI) for Davos 2021

Davos 2021	date	B.1.1.7	B.1.351	B.1.617.1	B.1.617.2	BA.1	BA.2	P.1	undetermined
Point estimate	05/11/2021	0.028447596	0.002367789	0.044378929	0.838073406	0.021142663	0.00450093	0.002589447	0.058499241
	12/11/2021	0.034517818	0.005381831	0.042535976	0.817203742	0.030626931	0.005485472	0.003010871	0.061237358
	19/11/2021	0.037569153	0.007979925	0.040458999	0.805056391	0.0379927	0.006038136	0.003210812	0.061693885
	26/11/2021	0.038844425	0.010240886	0.038212863	0.797531197	0.044685699	0.006345157	0.003279762	0.060860012
	02/12/2021	0.042070247	0.01264437	0.035425329	0.783854476	0.054415511	0.006952458	0.003482966	0.061154643
	03/12/2021	0.035760849	0.012981501	0.03091814	0.786881635	0.065875311	0.006227259	0.003262151	0.058093154
	05/12/2021	0.021790816	0.012122647	0.027263961	0.809485101	0.071938031	0.004402682	0.003092111	0.049904651
	07/12/2021	0.006840902	0.010227613	0.023629141	0.831081534	0.085339793	0.002737025	0.002743548	0.037400442
	09/12/2021	0.002526307	0.00846591	0.021464631	0.813074086	0.117352361	0.00293074	0.002723708	0.031462257
	10/12/2021	0.003254512	0.006738624	0.019895302	0.767229959	0.167151039	0.003674964	0.002742249	0.02931335
	12/12/2021	0.00458344	0.004891258	0.017585908	0.706838708	0.234582916	0.003915962	0.002623576	0.024978232
	14/12/2021	0.00731957	0.003401118	0.013918778	0.614988128	0.338424419	0.003599427	0.001885549	0.016463012
	19/12/2021	0.010106208	0.0018141	0.010151216	0.521662391	0.444222528	0.003324702	0.001011918	0.007706938
	21/12/2021	0.012952014	0.000176431	0.006319963	0.424566468	0.552975557	0.002954192	5.53753E-05	0
	23/12/2021	0.015840963	0	0.002447136	0.318757264	0.660533193	0.002421444	0	0
Upper 95% CI	05/11/2021	0.036326476	0.022812516	0.12095502	0.906957282	0.030045205	0.02256225	0.00859992	0.135244632
	12/11/2021	0.043968501	0.030702046	0.109729255	0.880281605	0.04030897	0.027450052	0.007924917	0.120163106
	19/11/2021	0.04775245	0.035288332	0.089328347	0.859633618	0.04805454	0.030180385	0.007096781	0.104909447
	26/11/2021	0.049283845	0.037935311	0.079637208	0.84031491	0.05553788	0.031707813	0.006889482	0.093297437
	02/12/2021	0.053358265	0.043437967	0.070466732	0.82537084	0.067604522	0.034375197	0.007530133	0.089731333
	03/12/2021	0.045118034	0.039702714	0.058104905	0.829260668	0.081435612	0.030509348	0.007119719	0.093259869
	05/12/2021	0.027240871	0.029310781	0.058140912	0.849002425	0.085625983	0.019243291	0.010967004	0.093384111
	07/12/2021	0.011613012	0.024708446	0.055968016	0.872910813	0.097276536	0.006402613	0.008845622	0.082216981
	09/12/2021	0.008977753	0.022449403	0.058443001	0.860592504	0.137770544	0.007270914	0.008794982	0.076830257
	10/12/2021	0.010698073	0.016959362	0.054094142	0.81812068	0.192174696	0.009553159	0.009004741	0.076077639
	12/12/2021	0.014174675	0.012882521	0.04653713	0.757435851	0.267296623	0.011013488	0.008764563	0.066059634
	14/12/2021	0.023736209	0.009800132	0.033379735	0.655562123	0.37715767	0.010037493	0.006359558	0.044330894
	19/12/2021	0.034983037	0.00614494	0.024410781	0.565555465	0.48824034	0.009642878	0.003477888	0.023932565
	21/12/2021	0.048699503	0.002208943	0.021727128	0.482932063	0.602010933	0.008965501	0.000299651	0.013343888
	23/12/2021	0.064122169	0.001364505	0.020367393	0.408954319	0.724121861	0.010213639	4.69408E-05	0.006327927
Lower 95% CI	05/11/2021	0.021846857	0	0.001843377	0.689529172	0.013647124	0.000245544	0.00029866	0.023015054
	12/11/2021	0.026630332	0	0.0062561	0.704922853	0.022220643	0.000490881	0.000370185	0.032532057
	19/11/2021	0.028919919	0.001100764	0.009513795	0.716339763	0.029524026	0.000674115	0.000473157	0.03820874
	26/11/2021	0.029854802	0.001718249	0.011421383	0.731231657	0.035050512	0.000861127	0.000464706	0.042253784
	02/12/2021	0.032359734	0.002401696	0.012505179	0.738864766	0.043140442	0.000837237	0.000490109	0.042580517
	03/12/2021	0.02725256	0.002563578	0.010571314	0.74688758	0.054117151	0.000699856	0.000594637	0.037009383
	05/12/2021	0.016213389	0.002823563	0.007449077	0.765244829	0.061636525	0.000716351	0.000461642	0.024405091
	07/12/2021	0.004412856	0.001693717	0.005156799	0.780829048	0.07147969	0.000509409	0.000261062	0.008998655
	09/12/2021	0.000147919	0.000969291	0.005264786	0.757926814	0.098652859	0.000231335	0.000120014	0.002763311
	10/12/2021	0.000156827	0.000623646	0.003817669	0.711865697	0.146588133	0.000271445	9.35152E-05	0.001252146
	12/12/2021	0.000290534	0.000374688	0.002700134	0.65666618	0.210761849	0.000245657	7.97641E-05	0.000128341
	14/12/2021	0.000379538	0.00017656	0.00161226	0.57614371	0.309299661	0.000269886	5.47303E-05	5.15254E-05
	19/12/2021	0.000115144	0	0.000550479	0.474677426	0.402292193	4.79199E-05	2.03867E-05	0
	21/12/2021	0	0	0	0.372021827	0.501532979	0	0	0
	23/12/2021	0	0	0	0.259139376	0.599354394	0	0	0

Table S3: Point estimates with upper and lower 95% confidence intervals (CI) for Landquart 2021

Landquart 2021	date	B.1.1.7	B.1.351	B.1.617.1	B.1.617.2	BA.1	BA.2	P.1	undetermined
Point estimate	05/11/2021	1.89989E-05	0.010466189	0	0.977948457	0	0	0	0.011566355
	12/11/2021	0.000541828	0.008518234	0.087833549	0.855253321	0.00228179	0.000800299	0.000491591	0.044279389
	19/11/2021	0.068933663	0.017456626	0.129401596	0.578171924	0.071524115	0.010910436	0.010723736	0.112877903
	26/11/2021	0.102355065	0.02504963	0.098653086	0.51375962	0.104102579	0.015425057	0.015458485	0.125196477
	10/12/2021	0.068461597	0.023178738	0.036012786	0.706071909	0.068553968	0.010042496	0.01016812	0.077510387
	23/12/2021	0	0.007244851	0.016687035	0.976068114	0	0	0	0
Upper 95% CI	05/11/2021	0.000464043	0.032409955	0.0456059	1	0.000517686	0.000485317	0.000148101	0.058569059
	12/11/2021	0.001351626	0.026047031	0.182664921	0.955872129	0.002450305	0.002629931	0.001781611	0.140702912
	19/11/2021	0.088884559	0.067558145	0.277938153	0.698979443	0.089706604	0.056842333	0.055429422	0.253508585
	26/11/2021	0.132553098	0.10109208	0.227986562	0.627524587	0.133560877	0.084412284	0.082442989	0.225036082
	10/12/2021	0.088723292	0.073798136	0.077196188	0.755788175	0.08892811	0.055974353	0.055071482	0.106637564
	23/12/2021	0	0.029300838	0.083216188	1	0	0.000109351	4.71507E-05	0.039321407
Lower 95% CI	05/11/2021	0	0.000194263	0	0.898412569	0	0	0	0
	12/11/2021	6.70136E-05	0.000495579	0.013183847	0.717674775	7.094E-05	4.19746E-05	3.20719E-05	0.003860322
	19/11/2021	0.048173639	0.001749863	0.02151538	0.423912614	0.048105945	0.001099741	0.0010605	0.06492774
	26/11/2021	0.071256146	0.002745553	0.01564885	0.416317326	0.071443068	0.001431991	0.001268937	0.086349913
	10/12/2021	0.047580769	0.00227526	0.006197404	0.662794945	0.047895934	0.000772508	0.000766073	0.0548665
	23/12/2021	0	0	0	0.98001646	0	0	0	0

Table S4: Point estimates with upper and lower 95% confidence intervals (CI) for Lostallo 2021

Lostallo 2021	date	B.1.1.7	B.1.351	B.1.617.1	B.1.617.2	BA.1	BA.2	P.1	undetermined
Point estimate	05/11/2021	9.01175E-05	0.000283894	0.037182934	0.936549433	0.000220608	0.000379037	0.000281442	0.025012534
	19/11/2021	0.00030839	0.000230512	0.021028984	0.961457566	0.00028498	0.00036481	0.000332096	0.015992662
	26/11/2021	0.000410209	0.000344391	0.018928887	0.960937542	0.000416163	0.000328987	0.000303872	0.01832995
	03/12/2021	0.000495044	0.00064798	0.02598627	0.938997268	0.000570437	0.000266133	0.000326362	0.032710508
	10/12/2021	0.000452172	0.000717792	0.028634148	0.931013549	0.000858459	0.000319463	0.000401556	0.037602862
	23/12/2021	0.000422994	0.000523291	0.022429937	0.949913596	0.00140717	0.000408842	0.000360037	0.024534132
Upper 95% CI	05/11/2021	0.000317524	0.000659595	0.136550321	0.991395612	0.000717152	0.00097347	0.000984039	0.06572646
	19/11/2021	0.000798518	0.000445909	0.059639444	0.989459564	0.000687435	0.000750521	0.000892957	0.042410625
	26/11/2021	0.001084369	0.000735887	0.043716515	0.988121548	0.000893344	0.000630913	0.000751229	0.04630467
	03/12/2021	0.001075653	0.001545132	0.068225991	0.984939774	0.001107613	0.000594311	0.000700237	0.078464791
	10/12/2021	0.001066541	0.001925247	0.07188288	0.980430834	0.001710738	0.000667094	0.000848264	0.098345777
	23/12/2021	0.000840261	0.001294626	0.065362853	0.991030159	0.00374193	0.001290449	0.000870272	0.083195901
Lower 95% CI	05/11/2021	0	0	0.002471801	0.806399698	0	4.06631E-05	1.59713E-05	0.00237007
	19/11/2021	9.17114E-05	5.65275E-05	0.003964809	0.915205526	5.93317E-05	0.000104512	6.11364E-05	0.003730224
	26/11/2021	0.000111631	5.31E-05	0.003782043	0.917050411	0.000106888	0.000104341	9.23847E-05	0.00465947
	03/12/2021	0.000153961	0.000134006	0.004631282	0.869960271	0.000181425	7.02673E-05	7.71539E-05	0.004200971
	10/12/2021	0.000127296	0.000188594	0.006146682	0.84644328	0.000266095	9.65081E-05	0.000102427	0.005692646
	23/12/2021	8.16785E-05	1.00702E-05	0	0.887474378	0.000252282	7.62296E-06	1.85263E-05	0.00048678

Table S5: Point estimates with upper and lower 95% confidence intervals (CI) for S-chanf 2021

S-chanf 2021	date	B.1.1.7	B.1.351	B.1.617.1	B.1.617.2	BA.1	BA.2	P.1	undetermined
Point estimate	05/11/2021	0.026211437	0.007078053	0.039007927	0.854382844	0.01160081	0.004695118	0.003616846	0.053406964
	12/11/2021	0.032121434	0.009889064	0.041718711	0.821803906	0.024612982	0.006185777	0.004403434	0.05926469
	19/11/2021	0.035239769	0.011829214	0.043124016	0.799308342	0.036511427	0.007194602	0.004819881	0.06197275
	26/11/2021	0.03673544	0.013175004	0.044035047	0.781330805	0.048695953	0.007962529	0.005017929	0.063047294
	02/12/2021	0.040093051	0.014914052	0.045224523	0.755184273	0.064351314	0.009167283	0.005447451	0.065618053
	03/12/2021	0.034554972	0.013435792	0.041017324	0.76412656	0.076168547	0.008844654	0.004644271	0.057207879
	05/12/2021	0.021847632	0.009606395	0.033047631	0.806311913	0.078300997	0.007071621	0.002871896	0.040941914
	07/12/2021	0.008687813	0.004943212	0.024938417	0.840463105	0.087735181	0.005664039	0.000964316	0.026603918
	09/12/2021	0.007202727	0.003185584	0.020572806	0.819317876	0.112668611	0.006649223	0.000395313	0.03000786
	10/12/2021	0.01101418	0.003716414	0.019665567	0.771101331	0.144170537	0.007856202	0.000473226	0.042002543
	12/12/2021	0.014058873	0.004823697	0.018064056	0.718288523	0.187706362	0.007886549	0.000568187	0.048603753
	14/12/2021	0.01547288	0.005669377	0.015635935	0.647306371	0.262080995	0.006966199	0.000635651	0.046232592
	19/12/2021	0.01707022	0.006609734	0.013467474	0.573877952	0.336850163	0.006132848	0.00070035	0.045291259
	21/12/2021	0.018597255	0.007684878	0.011595324	0.498152137	0.414216667	0.005127462	0.000770314	0.043855964
	23/12/2021	0.019762784	0.008691415	0.009793505	0.420501725	0.496158585	0.003883277	0.000835752	0.040372957
Upper 95% CI	05/11/2021	0.034438235	0.026669631	0.111402188	0.906167573	0.022178887	0.022466163	0.019291624	0.094354256
	12/11/2021	0.04201803	0.033152689	0.103959555	0.872357869	0.035270691	0.027179753	0.02334603	0.100061208
	19/11/2021	0.045823884	0.036577947	0.094766931	0.848709672	0.047057554	0.029875135	0.025372578	0.099941092
	26/11/2021	0.047872323	0.038525707	0.099221767	0.829804608	0.060810862	0.031809726	0.026199173	0.098866487
	02/12/2021	0.052929207	0.042084056	0.100168054	0.804915348	0.081377148	0.035499443	0.028332052	0.103633259
	03/12/2021	0.04714876	0.03585966	0.087280092	0.807520875	0.097175654	0.03191468	0.0238402	0.091332036
	05/12/2021	0.03299157	0.02282544	0.070655044	0.845560877	0.099783508	0.020562585	0.014090966	0.070737762
	07/12/2021	0.0165164	0.012716457	0.051282228	0.874733462	0.116497525	0.01384968	0.003597902	0.05100189
	09/12/2021	0.016973162	0.008302498	0.045633842	0.859378613	0.141091361	0.017019991	0.00087358	0.053904959
	10/12/2021	0.026722277	0.012258424	0.045996202	0.819603122	0.179983255	0.024204187	0.000962351	0.076180012
	12/12/2021	0.033206024	0.019595804	0.051941315	0.781119267	0.229722432	0.027504846	0.001680904	0.101842768
	14/12/2021	0.035678042	0.022330592	0.047142724	0.716735569	0.306647164	0.024143818	0.001910602	0.101378443
	19/12/2021	0.039655314	0.025299275	0.042122241	0.66689641	0.400374603	0.021990562	0.002043987	0.111674833
	21/12/2021	0.047722812	0.030644178	0.044550094	0.608722712	0.498042596	0.02078314	0.002074827	0.122803864
	23/12/2021	0.058179477	0.037108886	0.043439814	0.548670102	0.600022993	0.01781495	0.002383413	0.114127374
Lower 95% CI	05/11/2021	0.018718393	0	0.006305674	0.776434175	0.000692362	0	0.000277268	0.028616249
	12/11/2021	0.02282339	0.000743687	0.008353307	0.761740174	0.013616255	0	0.000379291	0.033907823
	19/11/2021	0.024810267	0.001200091	0.010081888	0.739976262	0.026408629	0.000792188	0.000456871	0.037995222
	26/11/2021	0.025612274	0.001267846	0.011776034	0.724004704	0.037658498	0.001078909	0.000535733	0.038551024
	02/12/2021	0.027727011	0.00139462	0.013102408	0.698352321	0.048886296	0.001212431	0.000626043	0.038649405
	03/12/2021	0.023407525	0.001425585	0.012529278	0.706474537	0.057538219	0.001139717	0.000597822	0.032918998
	05/12/2021	0.014065354	0.001051942	0.010325085	0.759223431	0.057648252	0.000932618	0.000449023	0.020759801
	07/12/2021	0.004314597	0.000728251	0.007607464	0.803907303	0.06131995	0.000673751	0.000266099	0.009619714
	09/12/2021	0.00100356	0.000702428	0.005070733	0.784700979	0.079410894	0.000376183	0.000128198	0.006617776
	10/12/2021	0.001517305	0.000527439	0.003282451	0.720671784	0.101819814	0.000500118	0.000126459	0.004857329
	12/12/2021	0.002022951	0.000342561	0.0025634	0.641409859	0.136829079	0.000321858	7.61691E-05	0.004798925
	14/12/2021	0.002155044	0.000371448	0.002470235	0.575650903	0.208262962	0.000232379	5.9682E-05	0.003664537
	19/12/2021	0.000939226	0.000154391	0.000711599	0.490707378	0.26462123	5.4258E-05	1.9171E-05	0.001018577
	21/12/2021	6.5411E-05	0	0	0.399039467	0.32251368	0	0	0
	23/12/2021	0	0	0	0.305809549	0.373544377	0	0	0

Table S6: Point estimates with upper and lower 95% confidence intervals (CI) for Davos 2022

Davos 2022	date	B.1.1.7	BA.1	BA.2	BA.2.75	BA.4	BA.5	undetermined
Point estimate	15/05/2022	0.000344358	0.000699301	0.373318031	9.85E-05	0.001933598	0.62322031	0.000385947
	19/05/2022	0.000518685	0.000115433	0.392309868	0.000382461	0.004076389	0.602324032	0.000273132
	22/05/2022	0.000517789	0.000105935	0.457002912	0.000698641	0.002545871	0.538806443	0.00032241
	24/05/2022	0.000472823	0.000148563	0.444845094	0.000727144	0.002938521	0.55043106	0.000436795
	26/05/2022	0.000442192	0.000340138	0.346227575	0.000667817	0.011710025	0.640101612	0.000510642
	29/05/2022	0.000367648	0.000416119	0.31169061	0.000436307	0.009155776	0.677270162	0.000663377
Upper 95% CI	15/05/2022	0.000944989	0.002584754	0.42337054	0.000310396	0.001718008	0.637734864	0.001329112
	19/05/2022	0.001102252	0.000578555	0.417100839	0.001151177	0.011594178	0.631760879	0.001077523
	22/05/2022	0.001089342	0.000652254	0.467301784	0.00262995	0.002397859	0.550568662	0.001164212
	24/05/2022	0.00108171	0.000761413	0.462653722	0.002844134	0.002780944	0.583708066	0.001281134
	26/05/2022	0.001089016	0.001071937	0.373245867	0.002431261	0.05443433	0.653129861	0.00137731
	29/05/2022	0.001196228	0.001334861	0.324346719	0.001258815	0.029072975	0.691224578	0.001638023
Lower 95% CI	15/05/2022	1.45E-11	8.54E-22	0.360238782	5.95E-14	3.23E-34	0.575701413	8.98E-20
	19/05/2022	1.55E-05	4.07E-21	0.361938606	1.14E-05	1.03E-24	0.579514058	8.28E-19
	22/05/2022	8.82E-06	1.53E-24	0.437679086	1.73E-05	1.61E-31	0.530464514	2.10E-16
	24/05/2022	4.86E-06	1.88E-22	0.413005542	1.92E-06	4.86E-25	0.533804022	5.92E-20
	26/05/2022	5.41E-05	6.73E-18	0.321604091	1.11E-05	0.003046795	0.619931312	6.63E-13
	29/05/2022	1.45E-05	5.44E-17	0.291886403	6.13E-07	0.001421247	0.671687997	1.01E-11

Table S7: Point estimates with upper and lower 95% confidence intervals (CI) for S-chanf 2022

S-chanf 2022	date	B.1.1.7	BA.1	BA.2	BA.2.75	BA.4	BA.5	undetermined
Point estimate	15/05/2022	0.00024356	0.000385889	0.983136469	0.000845071	0.006418389	0.008326704	0.000643918
	19/05/2022	0.000254764	0.000547458	0.637776023	0.000873411	0.003174132	0.356390615	0.000983597
	22/05/2022	0.000271836	0.000285199	0.647658879	0.000843472	0.003206047	0.346989949	0.000744617
	24/05/2022	0.000249723	9.46E-05	0.646993457	0.00081681	0.003259796	0.347911593	0.000674039
	26/05/2022	0.000232938	4.01E-05	0.595135906	0.000825841	0.003548373	0.399530176	0.000686698
	29/05/2022	0.000208333	1.38E-05	0.40495019	0.000865321	0.009780664	0.583315584	0.000866136
Upper 95% CI	15/05/2022	0.000940543	0.001471603	0.994160616	0.003337874	0.007686983	0.01330372	0.001658553
	19/05/2022	0.001116522	0.001512111	0.654083565	0.00353963	5.56E-05	0.361493872	0.001922123
	22/05/2022	0.001048268	0.000837037	0.675905563	0.003420735	0.000162323	0.358795044	0.001633715
	24/05/2022	0.000974695	0.000471781	0.675764292	0.003366461	0.000690836	0.359874364	0.001549355
	26/05/2022	0.000944444	0.000360978	0.654446848	0.003397596	0.003640898	0.43674252	0.001547191
	29/05/2022	0.001006044	0.00013397	0.484648132	0.003600573	0.030140954	0.617313047	0.001890724
Lower 95% CI	15/05/2022	3.34E-13	6.16E-16	0.981836978	3.27E-06	7.92E-21	0.003013487	3.28E-13
	19/05/2022	5.16E-07	5.63E-17	0.634538773	3.16E-06	1.59E-23	0.343847728	5.88E-10
	22/05/2022	1.37E-08	4.21E-19	0.638463924	8.78E-08	3.38E-37	0.322026986	6.24E-10
	24/05/2022	5.33E-12	8.91E-27	0.636498452	9.21E-09	3.10E-38	0.323392084	3.69E-11
	26/05/2022	1.58E-09	8.90E-29	0.558976946	1.77E-08	1.80E-38	0.343293654	5.78E-15
	29/05/2022	8.91E-09	4.78E-24	0.365876679	3.68E-07	1.06E-25	0.502233236	4.00E-09

Table S8: Point estimates with upper and lower 95% confidence intervals (CI) for Davos 2023

Davos 2023	date	BA.2.75	BA.5	BQ.1.1	XBB	XBB.1.16	XBB.1.5	XBB.1.9	undetermined
Point estimate	12/01/2023	0.000230169	0.113007084	0.000315163	0.216537069	0.000855895	0.431516962	0.200901425	0.036636233
	15/01/2023	0.002464405	0.024096953	0.001096552	0.274655815	0.008530515	0.515699197	0.151599572	0.02185699
	17/01/2023	0.038339777	0.003454989	0.002584829	0.29422457	0.013335362	0.541296737	0.061915232	0.044848503
	20/01/2023	0.308072014	0.006288268	0.003217171	0.223190193	0.002474282	0.348454737	0.00413054	0.104172795
	22/01/2023	0.440092243	0.014683608	0.002620435	0.228158344	0.001989779	0.204599797	0.000696571	0.107159221
	24/01/2023	0.381707906	0.009251489	0.002553874	0.276042539	0.002404758	0.221359505	0.003230415	0.103449514
Upper 95% CI	12/01/2023	0.003761042	0.821816535	0.001708297	0.942040968	0.005792595	0.982411862	0.499698984	0.511547564
	15/01/2023	0.026825323	0.175177414	0.003810971	0.990246137	0.094124005	0.99100359	0.492210677	0.217477492
	17/01/2023	0.490985562	0.017667833	0.006938036	0.99244281	0.189730132	0.992057197	0.202131769	0.486360861
	20/01/2023	0.991632576	0.014098983	0.007617754	0.990811382	0.00688301	0.991110797	0.005539514	0.976498342
	22/01/2023	0.993654194	0.021138235	0.006706212	0.987198306	0.006112312	0.987847574	0.002628828	0.981105406
	24/01/2023	0.991150986	0.059563075	0.006677866	0.987678775	0.007308523	0.980392694	0.003010552	0.976423942
Lower 95% CI	12/01/2023	5.31E-35	0.000531007	7.33E-34	1.77E-25	4.74E-22	1.77E-16	0.000255392	9.06E-34
	15/01/2023	4.56E-27	1.03E-11	6.09E-19	4.28E-23	9.50E-11	4.14E-17	0.000685523	2.08E-25
	17/01/2023	5.15E-18	1.99E-16	0.000424485	2.24E-25	9.63E-05	5.36E-18	0.000560736	4.19E-22
	20/01/2023	0.00131104	5.03E-20	0.000774068	3.09E-26	0.000366381	5.63E-21	2.54E-19	1.28E-22
	22/01/2023	0.001433565	1.68E-17	0.000608953	4.05E-28	0.000311035	1.12E-22	4.04E-17	4.77E-22
	24/01/2023	0.001230013	5.27E-11	0.000476459	6.18E-31	0.000259136	5.88E-22	1.23E-21	2.57E-26

Table S9: Point estimates with upper and lower 95% confidence intervals (CI) for S-chanf 2023

S-chanf 2023	date	BA.2.75	BA.5	BQ.1.1	XBB	XBB.1.16	XBB.1.5	XBB.1.9	undetermined
Point estimate	12/01/2023	0.000503581	0.008389914	1.92E-05	0.409840759	0.000234664	0.558322704	0.010482853	0.012206342
	15/01/2023	0.011580625	0.001604177	9.79E-05	0.243263237	0.002641907	0.707369481	0.026443743	0.006998929
	17/01/2023	0.022402374	0.001177471	0.000130621	0.246615745	0.001140909	0.710348312	0.011756301	0.006428267
	20/01/2023	0.016664474	0.000784096	7.69E-05	0.242007346	0.000221536	0.720976203	0.008591297	0.010678194
	22/01/2023	0.011900794	0.000683992	2.51E-05	0.242407111	7.35E-05	0.730664323	0.00840499	0.005840185
	24/01/2023	0.000863384	0.000699741	8.45E-06	0.247960958	2.49E-05	0.741282645	0.008350653	0.000809246
Upper 95% CI	12/01/2023	0.005448676	0.023880216	0.000147129	0.501231921	0.002258673	0.96497031	0.008179823	0.060236529
	15/01/2023	0.03651653	0.006093644	0.000673113	0.499179426	0.012788454	0.995769026	0.138747582	0.021901849
	17/01/2023	0.275878789	0.004430494	0.00058389	0.50282695	0.005104968	0.997900193	0.015860425	0.008578317
	20/01/2023	0.029530385	0.003281992	0.000360332	0.499750538	0.000668027	0.997860099	0.001297169	0.005569348
	22/01/2023	0.014175756	0.003024095	0.000175888	0.499782823	0.000202627	0.998840628	0.000311049	0.005175852
	24/01/2023	0.004548461	0.002636947	6.49E-05	0.500694924	9.09E-05	0.999268919	0.000150284	0.00468377
Lower 95% CI	12/01/2023	1.14E-36	1.83E-06	3.75E-37	0.017026241	9.11E-07	0.330863405	1.92E-05	1.17E-34
	15/01/2023	2.70E-33	5.34E-06	7.53E-36	2.74E-05	6.01E-06	0.447240311	2.81E-06	2.12E-35
	17/01/2023	4.50E-26	1.38E-05	1.48E-26	3.79E-11	1.25E-05	0.373778963	8.32E-06	5.27E-27
	20/01/2023	1.11E-15	5.39E-06	6.72E-24	1.80E-22	1.34E-05	0.403559305	7.16E-06	1.30E-23
	22/01/2023	5.03E-25	3.63E-06	1.54E-26	1.13E-28	7.11E-06	0.479401339	5.65E-06	5.51E-25
	24/01/2023	6.96E-39	1.53E-06	4.94E-31	1.50E-29	2.04E-06	0.493520318	6.22E-06	1.60E-30

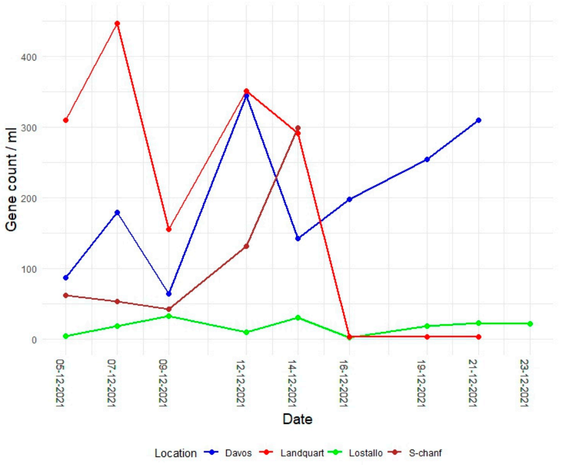

Figure S1. qPCR results of the wastewater samples testing for SARS-CoV-2 including Omicron in December 2021 in Davos (blue), Landquart (red), Lostallo (green) and S-chanf (brown).

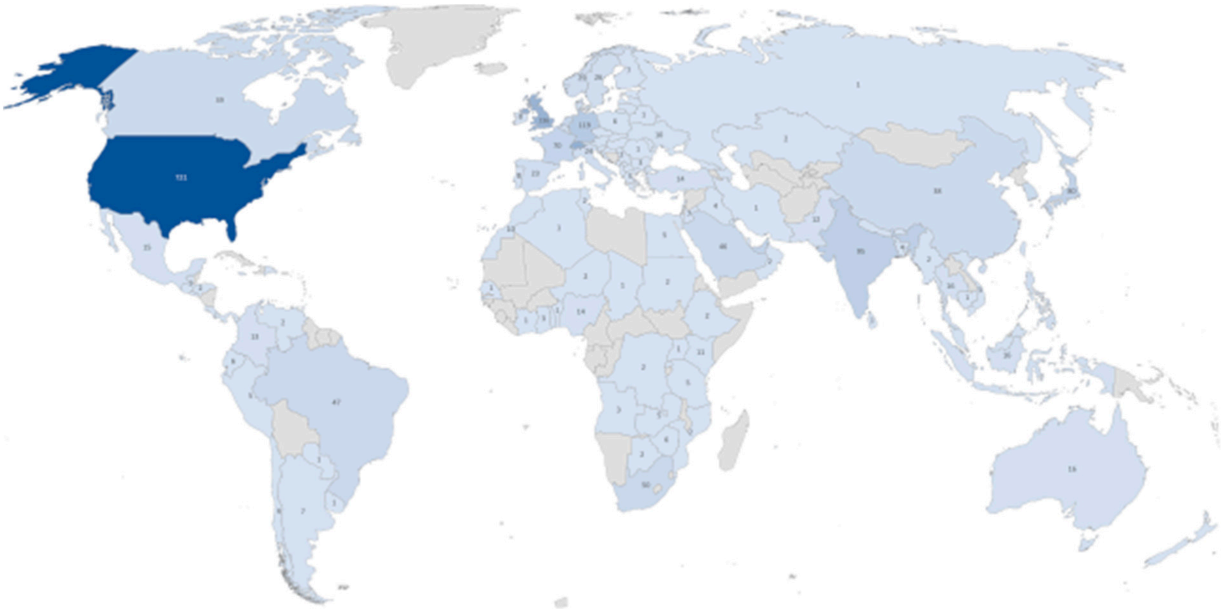

@Australian Bureau of Statistics, GeoNames, Geospatial Data Edit, Microsoft Crowdsourced Enrichments, Navinfo, OpenStreetMap, TomTom, Wikipedia, Zenrin

Figure S2. Number of WEF 2023 participants per country of origin; in blue the countries with participants, the darker blue, the more participants.
